# Expression Patterns, Activities and Carbohydrate-Metabolizing Regulation of Sucrose Phosphate Synthase, Sucrose Synthase and Neutral Invertase in Pineapple Fruit during Development and Ripening

**DOI:** 10.3390/ijms13089460

**Published:** 2012-07-26

**Authors:** Xiu-Mei Zhang, Wei Wang, Li-Qing Du, Jiang-Hui Xie, Yan-Li Yao, Guang-Ming Sun

**Affiliations:** 1 Key Laboratory of Tropical Fruit Biology, Ministry of Agriculture, South Subtropical Crop Research Institute, Chinese Academy of Tropical Agricultural Science (CATAS), Zhanjiang 524091, Guangdong, China; E-Mails: asiazhang1975@163.com (X.-M.Z.); duliqing927618@163.com (L.-Q.D.); xiejhww@163.com (J.-H.X.); yanliyao78@163.com (Y.-L.Y.); 2 Department of Food Science, Lousiana State University, Baton Rouge, LA 70803, USA; 3 Laboratory of Plant Genetic & Breeding, Anhui Agricultural University School of Life Science, 130 Changjiang West Road, Hefei 230036, Anhui, China; E-Mail: wangweisys@163.com

**Keywords:** *Ananas comosusr*, carbohydrate metabolism, enzyme activties, gene cloning, transcript expression

## Abstract

Differences in carbohydrate contents and metabolizing-enzyme activities were monitored in apical, medial, basal and core sections of pineapple (*Ananas comosus* cv. Comte de paris) during fruit development and ripening. Fructose and glucose of various sections in nearly equal amounts were the predominant sugars in the fruitlets, and had obvious differences until the fruit matured. The large rise of sucrose/hexose was accompanied by dramatic changes in sucrose phosphate synthase (SPS) and sucrose synthase (SuSy) activities. By contrast, neutral invertase (NI) activity may provide a mechanism to increase fruit sink strength by increasing hexose concentrations. Furthermore, two cDNAs of *Ac-sps* (accession no. GQ996582) and *Ac-ni* (accession no. GQ996581) were first isolated from pineapple fruits utilizing conserved amino-acid sequences. Homology alignment reveals that the amino acid sequences contain some conserved function domains. Transcription expression analysis of *Ac-sps*, *Ac-susy* and *Ac-ni* also indicated distinct patterns related to sugar accumulation and composition of pineapple fruits. It suggests that differential expressions of multiple gene families are necessary for sugar metabolism in various parts and developmental stages of pineapple fruit. A cycle of sucrose breakdown in the cytosol of sink tissues could be mediated through both Ac-SuSy and Ac-NI, and Ac-NI could be involved in regulating crucial steps by generating sugar signals to the cells in a temporally and spatially restricted fashion.

## 1. Introduction

Fruit taste and quality in pineapple (*Ananas comosusr*) mainly depend upon such factors as sugars, organic acids, firmness, amino acids and aromatic compounds. Sucrose and its components, glucose and fructose synthesized in source tissues are one of the most important sugars, which are transported into sink tissues such as fruit, shoots and other tissues [1]. Sucrose serves an integral role as a source of carbon and energy for non-photosynthetic tissues, which is central to plant metabolism and the most dominant metabolite involved in fruit growth and development [2]. Until now, attempts to elucidate the changes in metabolism that lead to accumulation of sucrose had focused on sucrose-metabolizing enzymes like sucrose phosphate synthase (SPS, EC 2.1.4.14), sucrose synthase (SuSy, EC 2.4.1.13) and neutral invertase (NI, EC 3.2.1.26) [3,4].

With the full-length or fragment SPS cDNAs isolated from fewer than 20 plant species from GenBank database, some functional characteristics of SPS were studied both developmentally and in response to specific stimuli [5–7]. SPS had also been shown to be regulated posttranslationally by protein phosphorylation, binding to 14-3-3 proteins, and allosteric regulation [4]. Although there is a direct correlation between sucrose accumulation and SPS enzymatic activity, the mechanism of its regulation is unknown during fruit ripening [7].

Some researchers had reported that SuSy activity was correlated with the direction of reversible sucrose cleavage in plant sink tissues supplied with ample sucrose and with high demand from carbon biosynthetic and respiratory pathways, cellulose and callose synthesis and sink strength [8]. It plays a key role in sucrose breakdown for the generation of uridie diphosphate glucose (UDPG) and possible preservation of energy via uridine diphosphate (UDP) activation of the hexose moiety [9]. In many species, SuSy had also been identified repeatedly as playing a central role in modulating sink strength of plants [10].

To keep the balance of sucrose-sink during fruit growth and development, invertases catalyse the irreversible hydrolysis of sucrose to glucose and fructose. In plant cells, there are three invertase isoforms each having different biochemical properties and subcellular localisation. Soluble neutral invertase (NI) and acid invertase (AI) are located in the cytosol and vacuole, respectively, and insoluble extracellular invertase is bound to the cell wall. By contrast, the physiological roles of plant NIs have largely remained elusive until recently, and only limited and fragmentary information is available, mainly because of low enzyme activity and gene expression levels [11].

In our previous study, a *Ac-SPS* gene coding pineapple SPS protein was isolated and might play a key role in sucrose accumulation during the fruit development [7]. Although the importance of sucrose metabolism in plant development through the generation of sugar signals had been reviewed [8], analysis of function and expression of three enzymes had been limited, especially in sucrose- or hexose-storing fruits such as pineapple. In this study, differences in carbohydrate contents and activities of the related enzymes were monitored to evaluate changes in sugar metabolism of different pineapple parts throughout fruit development. Moreover, three cDNAs coding *Ac-susy*, *Ac-ni* and *Ac-sps* were isolated from the mRNA of pineapple fruit. A comparison of their molecular structures and phylogenic origin was presented here, and the relationship between sugar accumulation and gene differential expressions was also investigated in apical, medial, basal and core sections of pineapple fruits.

## 2. Results and Discussion

### 2.1. Soluble sugar Contents

Though fruit flavor is related to both taste (e.g., sugar and acid content) and aroma components, the main component dictating pineapple fruit eating quality is influenced by total sugar content as well as composition [12]. In this study, three major sugar (fructose, sucrose and glucose) contents of pineapple fruits were determined throughout development stages.

**Figure 1:**
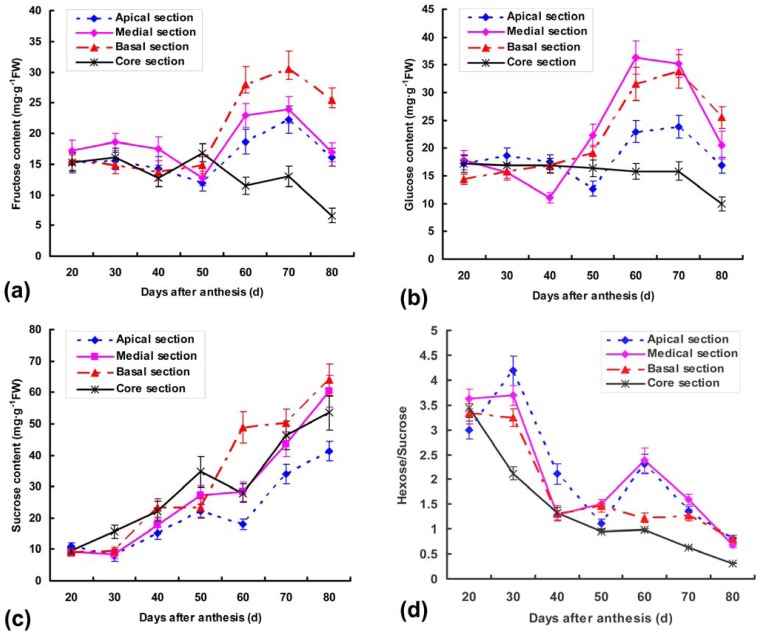
(**a**) Changes of fructose; (**b**) glucose; (**c**) sucrose contents; (**d**) sucrose/hexose ratio in the different fruit sections during various development stages. Each point is the mean of three determinations, and vertical bars are representative of ± S.E. (*n* = 3).

In the early stages of fruit growth, hexose (fructose and glucose) was the predominant sugar in nearly equal amounts except for medial section, accounting for *ca.* 80% of total soluble sugars (Figure 1a,b). A parallel gradual accumulation of glucose and fructose contents was detected in the apical and basal sections from 50 DAA to 70 DAA with the exception of core section, and then decreased in mature fruits. In medial sections, the fructose and glucose of medial sections also increased dramatically from 50 to 70 DAA and reached the highest levels at 70 DAA (23.86247 mg·g^−1^ FW) and 60 DAA (36.37503 mg·g^−1^ FW), respectively. Diametrically, the dynamic activities of two sugars are slightly decreased during fruit growth and maturation in core sections, which may be related to metabolizing-enzyme activities [13–15].

As reported by many researchers, sucrose is the chief contributor to sweetness, and is transported through sieve elements and can enter into sink organs directly by the plasmodesmata or the apoplastic space [15–16]. In our study, gradual increases of sucrose contents were detected starting from 20 DAA up to the end of fruit growth regardless of fruit sections, and reached 7 fold of initial content in basal section (Figure 1c,d). This may explain why sweet has a certain difference in the various portions and periods.

However, the variation tendency of sucrose was moderate in different parts of fruit between 50 and 60 DAA, accordingly, hexose (fructose and glucose) contents were sharply increased (Figure 1a,b). It indicated that the conversion of sucrose to hexoses was indispensable and may have other benefits. For example, hexose accumulation may function to increase the osmotic potential of the tissues and allow improved nutrient uptake [17]. Hexoses also play a role in regulating gene expression and therefore, accumulation in the vacuole would permit hexose storage without adverse effects on gene expression since the hexoses would be effectively excluded from the cytosol [17].

### 2.2. Isolation of *Ac-Sps*, *Ac-Ni* and *Ac-Susy* Genes and Sequence Analysis

#### 2.2.1. Multiple Alignments of Three Genes

The present study firstly isolated two cDNAs of *Ac-sps* and *Ac-ni* from pineapple fruits utilizing two pairs of degenerate primers according to the conserved domain of SPS and NI amino acid sequences from other plant species. Two 1131 bp and 1036 bp cDNA fragments were amplified by RT-PCR, and named *Ac-sps* (GenBank ID: GQ996582) and *Ac-ni* (GenBank ID: GQ996581), respectively. By alignment, the deduced SPS and NI polypeptides present high-level identities with corresponding regions of glucosyltransferases and neutral invertase from other plants (data not shown). In SPS amino acid sequences, the alignment analysis showed that *Ac-SPS* shared with amino acid identities of 85%, 84%, 82% and 81% to rice (*Oryza sativa*, AAQ56529) and green bamboo (*Bambusa oldhamii*, AAR16190), broomcorn (*Sorghum bicolor*, XP_002441522), wheat (*Triticum aestivum*, AAQ15106) and kiwifruit (*Actinidia deliciosa*, AAC39434), respectively.

In addition, the encoded NI polypeptide shared 94% identity with rice (*O. sativa*, EAY86114), 93% identity with broomcorn (*S. bicolor*, XP002452195) and grape (*Vitis vinifera*, XP002264960), 88% identity with Arabidopsis (*Arabidopsis thaliana*, NP197643), respectively. Interestingly, the amino acid homology of NI is more significant in the carboxyl terminal domain than in the amino terminal due to the fact that the catalytic domain is concentrated in the carboxyl terminal region within about 200 residues [11].

As expected, *Ac-SuSy* reported (GenBank ID: DQ438976) also showed a high degree of similarity to those of sucrose synthases from other species, and was thus classified among the large group of dicot *susy* genes (data not shown). The carboxyl terminal domain was more conserved than that of amino terminal. By contrast, the deduced amino acid has 87% homolog with rice (*O. sativa*, ABF95854) and maize (*Zea mays*, ACF78506), 86% homolog with broomcorn (*S. bicolor*, XP002465303) and 84% homology with soybean (*Glycine max*, XP003521575).

#### 2.2.2. Domain Characteristics and Phylogenetic Analysis of *Ac-SPS*

**Figure 2:**
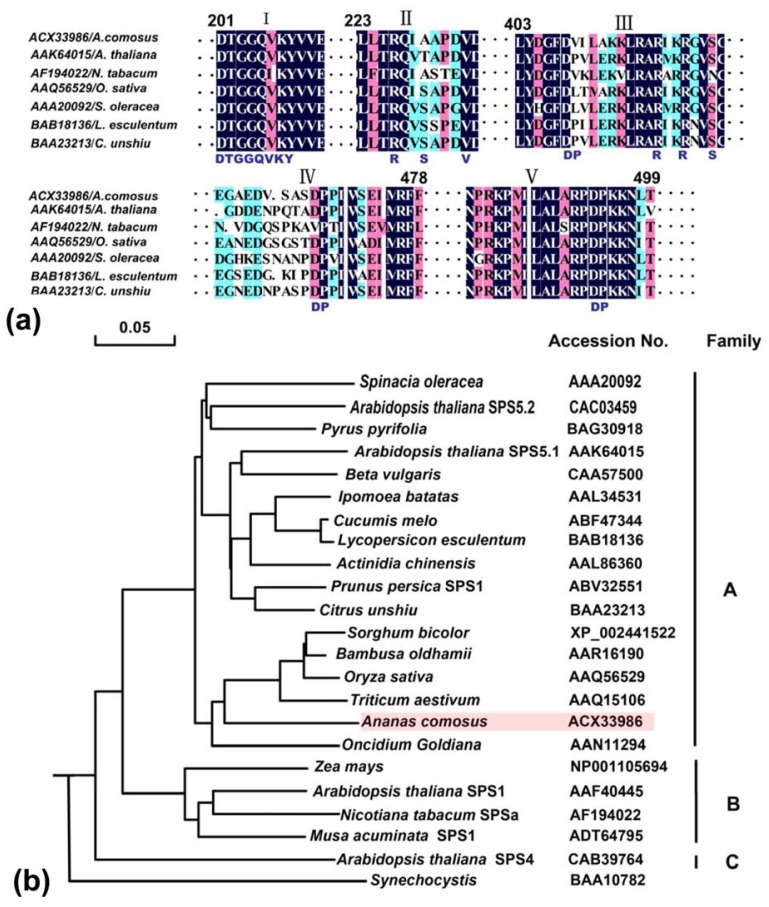
(**a**) Conserved regions and phylogenetic analysis of sucrose phosphate synthase (SPS) protein sequences. (I) some considered function domains were uncovered as the putative Fru6P binding site; (II) the 14-3-3 regulated phosphoserine and uridie diphosphate glucose (UDPG) binding domain; (III) the osmotically regulated phosphoserine; (IV, V) various aspartate-proline pairs determined in either spinach or maize or both [19]. Each active site of the various motifs was highlighted in capital letters under the alignment. Numbering of amino acids is according to *S. oleracea*; (**b**) the amino acid sequences of the ORFs were initially aligned and tree was calculated by Neighbor-Joing algorithms and rooted with *Synechocystis*. The GenBank accession numbers and name of the sequences were showed.

By alignment of the SPS amino acid sequences, some functional domains are highly conserved, which are mostly located in the *N*-terminal half of the protein including the glucosyltransferase and SPP-like domains (Figure 2a). The Fru6P binding region (I) is identical in all sequences with the exception of an isoleucine substitution for valine in SPS2 of *Nicotiana tabacum*, The regulatory phosphoserine (SPS-229) involved in 14-3-3 protein and UDPG binding sites have not been conserved (II), but the suggested recognition elements for the protein kinase are also nearly identical in all sequences. SPS is modulated by multisite protein phosphorylation in response to light, osmotic stress (SolSer-424), nitrogen supply, and temperature and also binds 14-3-3 proteins (SolSer-229) [18].

A phylogenetic analysis of plant *sps* genes had been reported previously within the broader A, B, and C families, according to the classification of Langenkämper *et al*. (2002) [19]. Although only one member (AtSPS4) of the C family was represented in the full-length sequences, A and B-families had obvious internal splits between the dicot and the monocot in our study. Up to now, none of the known monocot SPS genes belongs to the C family because of the relative scarcity of SPS sequences from monocots. Family A has 16 members with well-supported internal splits between the 11 dicotyledonous sequences (subfamily a1) and the six monocotyledonous sequences (subfamily a2). B family has four members containing two monocotyledons and two dicotyledons, respectively. Though the *Ac-SPS* from *A. comosus* is seemed to be allied with the A family, it is somewhat more divergent than the rest (Figure 2b). It seemed clear that these three gene families must have arisen before the separation of the monocots and dicots, which was thought to have occurred about 200 million years ago [20]. Furthermore, Lunn (2002) also found that the conservation of the exon-intron splice sites within the A, B and C family genes in Arabidopsis, rice and maize were consistent with all three families having a common origin [21].

#### 2.2.3. Domain Characteristics and Phylogenetic Analysis of Ac-NI

**Figure 3:**
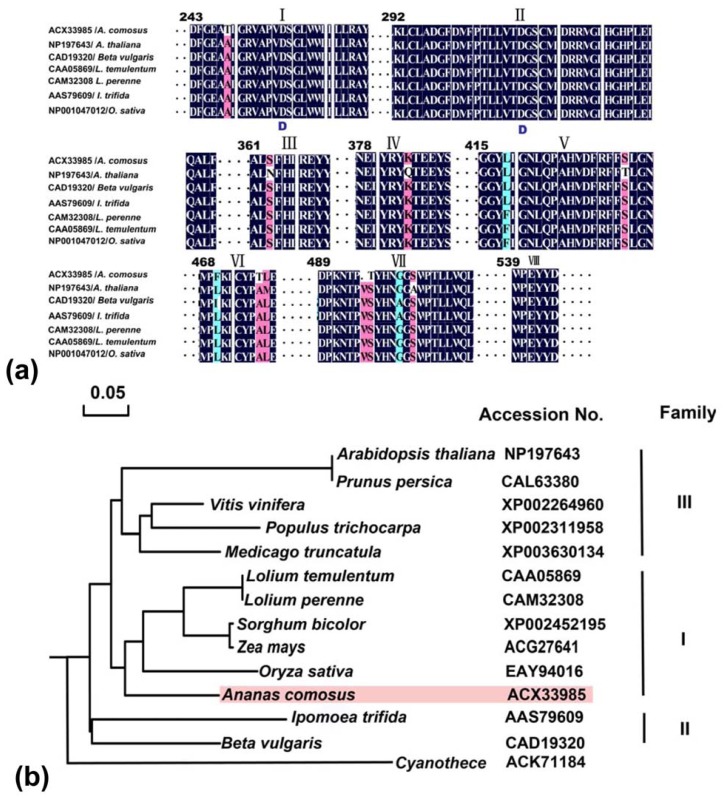
Conserved regions and phylogenetic analysis of, neutral invertase (NI) protein sequences. (**a**) Large regions of homology are indicated in boxes I-VIII. Putative catalytic sites (Asp^256^ and Asp^310^) are highlighted in I and II conserved regions, respectively. The numbers above the alignment represent the neutral invertases sequence of *L. temulentum*; (**b**) Phylogenetic dendrogram was generated by the multi-alignment using Molecular Evolutionary Genetics Analysis (MEGA) 4.0 based on identity and rooted with *Cyanothece*. Sequences are designated by accession numbers and name of organisms from GenBank database.

The eight conserved regions of the seven NIs identified from different plants were shown including the *A. comosus* in our study (Figure 3a). Two residues (Asp^256^ and Asp^310^) attributed to the enzyme active sites were proposed in I and II conserved regions of NI amino acids. Interestingly, this VII peptide domain was lack of Val^454^ and changed Ser^455^ with Thr in the conserved peptide sequence. However, whether the presence of high conserved domains plays a key role in regulating the sugar metabolism needs to await further analysis.

Thirteen full protein sequences of NI family from 12 organisms were identified from Genbank. Analysis of the evolution revealed that the NIs of higher plants were classified into the three clades by comparing the conserved function domains (Figure 3b). The Ac-NI belongs to the clade I from monocots, and is conserved among them sharing about 68%–93% identity between each other. The other two clades (II, III) are composed of dicots from *A. thaliana*, *Beta vulgaris* and *V. vinifera*, *etc.* Ac-NI protein is predicted to be localized in mitochondria, because all proteins from the common organelles are located in the same cluster divided into two subclusters, plastid- and mitochondria-localized types [22]. Vargas *et al*. (2003) suggested that NI homologues were restricted to cyanobacterial species and plants, and higher plant NIs might have originated from an orthologous ancestral gene after the endosymbiotic origin of chloroplasts [23]. Though this hypothesis could well explain the presence of plastid-localized form of neutral invertase, it was difficult to imagine that mitochondrial form in higher plants occurred from plastid-form by exchanging of targeting sequences to plastids and mitochondria.

#### 2.2.4. Phylogenetic Tree Analysis of Ac-SuSy

**Figure 4:**
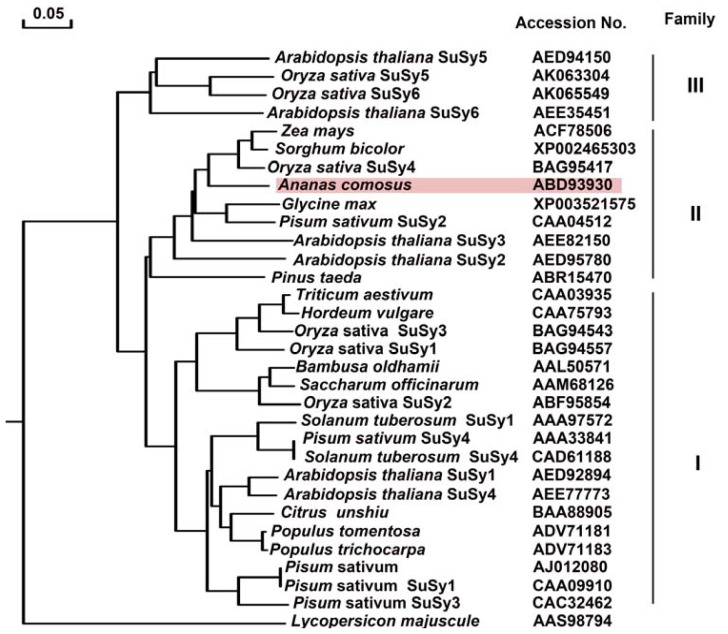
Phylogenetic dendrogram of the deduced amino acid sequences of pineapple SuSy gene constructed using the Neighbor-Joining method with MEGA software 4.0. Sequences were designated by accession numbers and name of organisms from GenBank database.

In order to explore a comprehensive analysis of evolutionary relationships among SuSy gene families between pineapple and other plant species, including the twelve isoforms of rice and Arabidopsis SuSy, a total of 31 plant and one bacteria SuSy amino acid sequences, representing 18 species, were aligned (Figure 4). The phylogenetic tree analysis revealed both relatively deep evolutionary root and the existence of more recent duplications for the *SuSy* genes. Using one *SuSy* from bacteria as the outgroup, the plant *SuSy* genes could be subdivided into three clearly distinct groups, including genes from angiosperms and gymnosperms. These groups were designated class I, class II and class III, respectively. For pineapple SuSy, it was fallen into this monocot-specific group II, and clusters together with other monocot genes by forming an independent clade to the exclusion of dicots genes. As shown in Figure 4, SuSys from dicot and monocotyledonous plants were found in all the three groups, especially, SuSy proteins in class I can be well classified into two distinct subclades. In addition, six rice SuSys, six Arabidopsis SuSys and four pea SuSys were also distributed in the dicot branch of classes I, II, and III (Figure 3). Those results suggested that SuSy evolutionary divergence originated before the common ancestor of dicots and monocots, and most second duplication events occurred before the monocot/dicot divergence.

### 2.3. Transcript Expression Analysis of *Ac-sps*, *Ac-susy* and *Ac-ni* genes

In order to evaluate the importance of carbohydrate-metabolizing enzymes throughout pineapple fruit development, the spatial and temporal expression patterns revealed that three genes were differentially expressed in immature and mature fruits. 18S rRNA expression levels were chosen as standards for constitutive expression and gave closely similar results. Expression levels of *Ac-sps* mRNA revealed some differences in all developmental stages and different parts of pineapple fruits.

**Figure 5:**
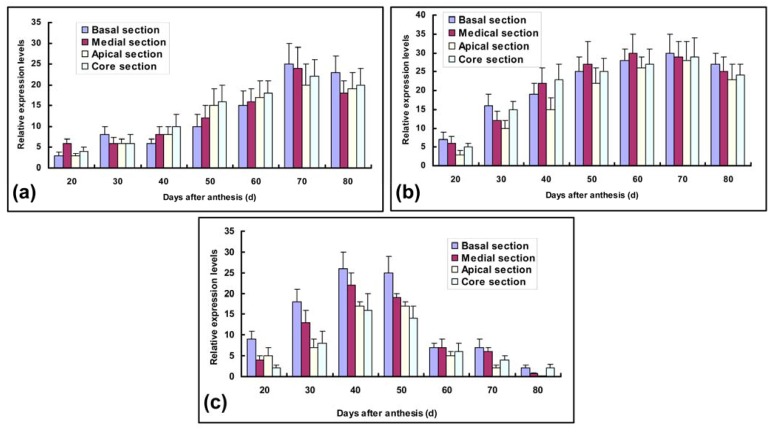
Transcript expression analysis of (**a**) *Ac-sps*; (**b**) *Ac-susy*; (**c**) *Ac-ni* genes during fruit development using quantitative PCR.

Recent evidence suggested that SPS may play a more important role in sucrose synthesis and accumulation [7,15,17,19], as supported by the transcript characteristics of *Ac-sps* in sucrose accumulation (Figure 5a). In contrast to mRNA levels during the initial phase of anthesis, *Ac-sps* transcript levels of fruit different sections were nearly devoid in 20 DAA except for medial parts, which was associated with low levels of sucrose in immature fruit sections, as also observed by Botha & Black (2000) [24]. They gradually reached two peaks of accumulation with maxima at 30 and 70 DAA in basal and medial sections, respectively, and then were down-regulated up to 80 DAA. However, a transcript peak of 30 DAA was lost in apical and core sections. This reason may be caused by the differences of organic acid and secondary metabolites in various fruit sections [17]. Experiments with banana had consistently demonstrated SPS activity increase with accumulation of sucrose in agreement with our results [25]. Strand *et al*. also provided the evidence that decreased expression of *sps* can inhibit sucrose synthesis in transgenic *A. thaliana* [26]. Though a remarkable rise is showed along with fruit maturation, there is a significant difference between the core and apical sections and the basal and medial sections (*p* < 0.05, *n* = 3) throughout the ripening process of pineapple fruits. Zhang *et al.* (2010) also found that different harvest seasons had serious influence on the activities of carbohydrate-metabolizing enzymes and sugar component in the different stages of fruit development [15].

The other most significant contributions of our study are that not only SPS but also SuSy and NI activities are positively correlated with sugar accumulation. Though SPS activity had also been implicated in sugar metabolism, the roles of other two enzymes had been less clear [11]. In our experiments, *Ac-susy* transcription levels were gradually up-regulated, and exhibited obvious profiles in the different sections with fruit development (Figure 5b). Thereafter, *Ac-susy* transcripts in four different sections showed a marked decrease to basal levels after 70 DAA until harvest. In medial and basal sections, there are more obvious than the other parts in *Ac-susy* transcript levels. However, some studies had attributed sucrose breakdown roles to these two latter enzymes [16], but our study clearly indicated that activities of SuSy were increased with sucrose accumulation in different sections of pineapple fruits, rather than the opposite, similar with results detected in watermelon [3] as well as other fruit [27]. These results indicated *Ac-susy* transcript was bound up with sucrose increase. Ciereszko et al. also demonstrated that the sucrose/glucose-dependent SuSy expression was strongly induced in transgenic Arabidopsis hexokinase-overexpressing plants [28]. Although details of this regulation are unclear, SuSy activity and expression of the corresponding gene(s) were more or less related with hexokinase.

Due to low enzyme activity and gene transcription levels, NI was scarcely studied [11]. Nevertheless, higher levels of the *Ac-ni* transcripts were detected between 30 DAA and 50 DAA with a transient increase of hexose contents in our study (Figure 5c). On the other hand, though the abundance of *Ac-ni* mRNA also increased and had a single peak around 40 DAA regardless of different sections in the early development of the fruits, *Ac-ni* expression levels decrease come up more early than that of *Ac-susy* and *Ac-sps*. From 40 to 80 DAA, *Ac-ni* transcription was down-regulated to a negligible level, especially in the core and basal sections. These data together suggest the putative role of hexose in regulating *Ac-ni* transcription through a negative feedback control had been evaluated. This hypothesis may be further supported by the *Ac-ni* transcript levels of different fruit sections, because hexose accumulation of the basal section analogous to *Ac-ni* transcript levels precedes the other parts. Nonis *et al.* (2007) also reported *Ac-ni* transcription levels can be improved using the hexokinase inhibitor NAG [29]. While previous studies on NI revealed small changes in gene expression among different tissues and during organ development [22], the pineapple NI studied in this article displayed a highly modulated pattern of expression in response to developmental clues. The major function of the high and constant transcription activity of *Ac-ni* in immature pineapple fruit is to maintain the cellular hexose concentrations. A decreased invertase activity would lead to increases in sucrose/hexose ratios [30,31]. This emphasizes not only the continuous hydrolysis of sucrose catalysed by invertase, but also the possibility of continuous sugar exchange between cytosol and vacuole (sucrose influx and hexose efflux).

### 2.4. Detection of Ac-SPS, Ac-SuSy and Ac-NI Activities

As expected, analogous results had been obtained in a comparison of enzyme activities and gene transcript levels. Those clearly indicated that sucrose accumulation occurs only in the presence of threshold activities of the three enzymes directly involved in sucrose metabolism. However, the dynamic patterns of three enzyme activities and transcripts during fruit development could not compare directly with each other, because multiple gene families could be differentially expressed in various parts and developmental stages of plants [18]. SPS, SuSy and NI had been reported in different flowering plants and database searches supported the existence of many isoforms of these enzymes [10,19,30]. The enzyme activity is synthetically performed by translations of many isozymes, but the mRNA transcript is one of the isozymes. Moreover, the protein contents of enzyme increased, and the increase of transcripts may push up the activity of enzymes.

**Figure 6:**
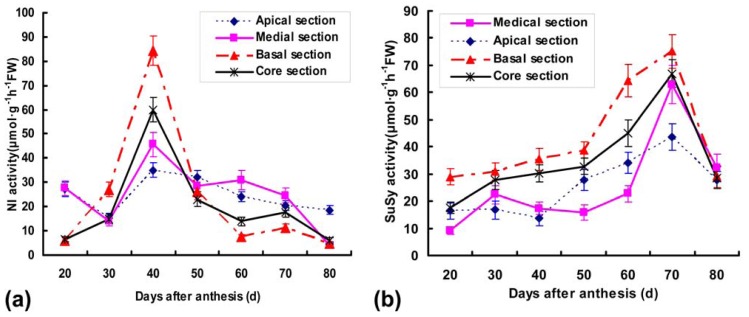

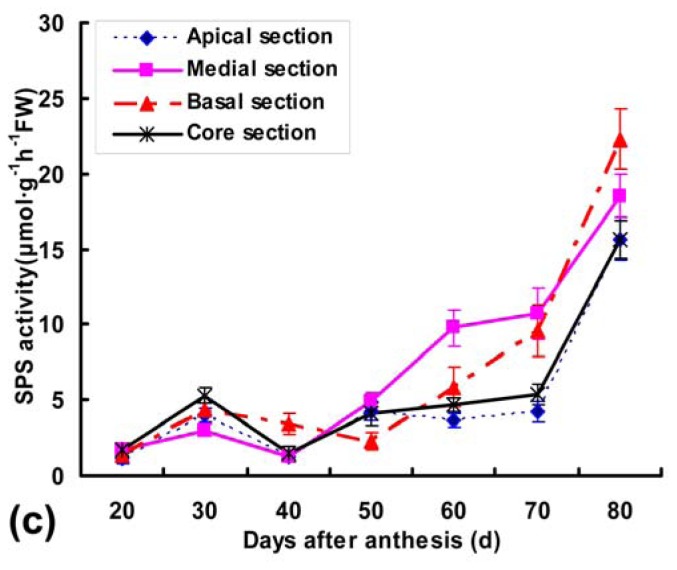
Activity profiles of (**a**) NI; (**b**) SuSy; (**c**) SPS in crude protein extracts of different fruit sections during pineapple development. Activities were expressed as μmol product synthesized g^−1^ h^−1^ FW. Each point is the mean of three determinations, and vertical bars are representative of ± S.E. (*n* = 3).

In some fruits, sucrose is translocated from the source to the fruit and is either apoplastically hydrolysed to fructose and glucose by invertase, or is cleaved symplastically to fructose and UDP-glucose by sucrose synthase [31]. In the present work, the Ac-NI activities were very low in basal and core sections at 20 DAA, but rapidly increased and reached maxima (14-fold and 10-fold, respectively) at 40 DAA. A pronounced increase was also detected in apical and medial activities from 30 to 40 DAA (Figure 6a). Thereafter, activities of various sections showed similar patterns with a slight peak at 70 DAA, followed by a gradual decrease towards becoming undetectable at 80 DAA. A positive correlation between sugar accumulation (Figure 1) and levels of SuSy and SPS activities (Figure 6b,c) were indicated in fruit different sections during development, which is paralleled with the transcript characteristics of two genes. These results suggest that sucrose synthase can only cleave the sucrose translocated to fruit tissue at a mature stage, and that NI activity is closely related to the accumulation of fructose and glucose in immature pineapple fruit. This is consistent with the suggestion that the two steps of sucrose degradation by SuSy and NI and the re-synthesis by SPS in fruit could generate a locally increased sucrose concentration gradient in the zone of phloem unloading, thus favoring sucrose import [17]. The potential capacity for re-synthesis of sucrose by the action of SPS in fruit is also supported with the presence of SPS activity and transcript of *Ac-sps*, which increase until the mature stage in fruit different sections (Figure 6c). A series of actions by SuSy and SPS during maturation could be important to sink strength in citrus fruit at this stage [5]. Thus, NI may have a role in supplying materials for growth and cell wall construction, whereas SuSy may supply SPS with substrates (UDP-glucose and fructose) for the re-synthesis of sucrose [9]. Substantial progress has been also made in studies on SuSy synthesizing UDPG which is essential for sucrose and cell wall biosynthesis [32]. Since pineapple fruits have a high capacity to cleave sucrose to glucose and fructose in immature stages (Figure 1), it seems likely that it is glucose rather than sucrose that serves as a signal in upregulating *Ac-susy* expression. Though the physiological role of NI and SuSy in sucrose metabolism is not completely clear in fruit development, the positive correlation with sucrose accumulation in our study suggests the need to rethink this step of sucrose metabolism in the context of sink sugar accumulation.

## 3. Experimental Section

### 3.1. Plant Materials

Field-grown pineapples (*A. comosus* cv. Comte de paris) were cultivated in the pineapple resource bank of South Subtropical Crops Research Institute. The experimental design used in this study was under the same management conditions such as irrigation, fertilization, soil management, disease control and pruning. Ninety fruit samples had been selected after the full florescence period from April to June in 2007. The fruits were randomly sampled every 10 days from the 20th day after anthesis (DAA) and cut transversely into apical, medial, basal and core sections, after the size and weight of crowns and fruits were measured. And then, the tissues were immediately frozen in liquid nitrogen and stored at −80 °C before being analyzed. These sliced fleshes of 30 fruits were pooled together as one of three replications at each harvesting time.

### 3.2. Plant Materials Determination of Sugar Content and Composition

For glucose, fructose and sucrose content, the sugars were extracted by grinding flesh tissues (10 g) in 80% ethanol, adjusted to pH 7.0 with 0.1 M NaOH, and heated for 5 min at 80 °C. The extract corresponding to 0.5 g fresh weight (FW) was dried in vacuum, redissolved in water and the solution was passed through an ion-exchange column (Dowex 50W-X8 and Dowex 1-X8). The eluate (10 μL) was then analyzed by high-performance liquid chromatography (HPLC) [13] (Shimadzu LC-6A; Kyoto, Japan) equipped with a RI detector and a SP1010 column (Showa Denko K. K., Tokyo, Japan) at a flow rate of 0.5 mL·min^−1^.

### 3.3. Cloning and Expression Analysis of *Ac-sps*, *Ac-susy* and *Ac-ni*

#### 3.3.1. Isolation of Total RNA and Sequence Amplification

Total RNA was extracted with Trizol Reagent (Invitrogen, Carlsbed, CA, USA) from different parts of pineapple fruits. The purity and integrity of total RNA were verified with a spectrophotometer at 230 nm, 260 nm and 280 nm (NanoDrop, Technologies Inc.) and 1.2% denaturing agarose gels (Qiagen, RNeasy Mini Handbook). For cloning of the genes related to sugar-metabolizing, SMAR™ PCR cDNA Synthesis Kit (BD Biosciences Clontech, United States) was used for the synthesis of cDNA starting from 0.5–1 μg of poly (A)^+^ RNA according to the instructions of the manufacturer.

Based on conserved amino acids from the available Genbank (http://www.ncbi.nlm.nih.gov/), two sets of degenerate primers and a pair of specific primers were designed for cloning *Ac-sps*, *Ac-ni* and *Ac-susy* genes, respectively (Table 1). PCR was carried out in a 25 μL reaction mixture containing 2.5 μL of reaction mixture 10× PCR Buffer II (Takara, Japan), 1.25 mM MgCl_2_, 4 mM dNTP/analog Mixture, 1 U ExTaq, 2 μL cDNA, 0.8 μM forward and reverse primers. The reaction mixture was overlaid with a drop of mineral oil and was allowed to run for 30 cycles (95 °C for 30 s, 52 °C, 54 °C and 55 °C for 30 s, 72 °C for 1.5 min for *Ac-sps*, *Ac-ni*, *Ac-susy*, respectively). PCR products were visualized on a 1.2 % agarose gel (*w*/*v*) including ethidium bromide (1 μg·mL^−1^), and then cloned into the pGEM-T Easy Vector System I (Promega, Madison, WI, USA) with T4 DNA ligase (Takara, Japan) according to the manufacturer’s instructions. Electro-transformation was used for *Escherichia coli* (JM109) with the Gene Pulser II system (Bio-Rad Laboratories Inc., Hercules, CA, USA) at 1500 V.

#### 3.3.2. Sequence Comparisons

All similarity searches were executed using blastn or blastx algorithms [33]. Multiple alignment and phylogenetic analysis were carried out using the Clustal W [34] and viewed by the Megalign (DNAstar Inc.). A phylogenetic tree was constructed by the Neighbor-Joining (NJ) method using the NJ algorithm implemented in the Molecular Evolutionary Genetics Analysis (MEGA) software version 4.0.

#### 3.3.3. Real-Time PCR Analysis of Gene Expressions

Quantitative PCR was performed by using the first strand cDNA as templates on a Lightcycler (Roche Diagnostics), with the Light Cycler Fast Start Reaction Mix MasterPLUS SYBR Green according to the manufacturer’s recommendations. Cycling conditions were as follow: 95 °C for 5 min, 40 cycles at 95 °C for 10 s, 60 °C for 10 s, and 72 °C for 30 s. All amplifications were normalized with an 18S rRNA gene to confirm equal amounts of RNA template. PCR parameters were analogous to above descriptions using specific primers (Table 1). The data shown represent means of values obtained from three independent biological replicates.

### 3.4. The Extraction and Activity Analysis of NI, SPS and SuSy

Sugar-metabolizing enzymes were prepared essentially in frozen flesh tissue as described by Zhang *et al.* [15]. Fruit material was homogenised in 10 mL of ice-cold buffer containing 50 mmol·L^−1^ Hepes-NaOH (pH 7.5), 0.5 mmol·L^−1^ Na-ethylenediaminetetraacetic acid (EDTA), 2.5 mmol·L^−1^ DTT, 3 mmol·L^−1^ diethyldithiocarbamic acid, 0.5% (*w*/*v*) bovine serum albumin (BSA) and 1% (*w*/*v*) insoluble polyvinylpyrrolidone (PVP). After centrifugation at 12,000 g for 20 min at 4 °C, supernatants were dialyzed for approximately 20 h against 25 mmol·L^−1^ Hepes-NaOH (pH 7.5) and 0.25 mmol·L^−1^ Na-EDTA and used as the crude soluble enzyme extract. The insoluble pellet was washed twice in homogenising medium and then incubated with shaking for 4 h in ice-cold homogenising medium containing 1 mol·L^−1^ NaCl.

NI activities were assayed in a final volume of 25 mL, containing 0.2 mL of dialyzed enzymatic extraction, 0.8 mL of reaction solution (pH 4.8 or 7.2, 0.1 mol·L^−1^ Na_2_HPO_4_, 0.1 mol·L^−1^ sodium citrate, 0.1 mol·L^−1^ sucrose for neutral invertase). The activities were measured by the quantity of reducing sugars released in the assay media with dinitrosalicylic acid [14].

Activities of SPS were assayed of using 0.15 mL of reaction medium and 0.2 mL of enzyme sample. The reaction medium is composed of 50 mmol·L^−1^ Mops-NaOH (pH 7.5), 10 mM MgCl_2_, 5 mmol·L^−1^ glucose-6-phosphate, 10 mmol·L^−1^ fructose-6-phosphate and 5 mmol·L^−1^ UDPG. After the mixture was incubated for 30 min at 37 °C, the reaction was stopped by adding 0.1 mL 30% (*w*/*v*) NaOH and kept in boiling water for 5 min. When cooled to room temperature, the resorcinol solution (12%, *v*/*v*) of 0.5 mL and HCl (12 mol·L^−1^) of 0.5 mL were added into the mixture and held in an 80 °C water bath for 10 min. Blank controls were obtained by adding the sterile water to the reaction medium containing resorcinol. The procedure for the SuSy assay was identical to that of SPS except for the reaction mixture, where fructose 6-phosphate or glucose 6-phosphate was replaced by contained 10 mmol·L^−1^ fructose.

### 3.5. Statistical Analysis

DPSv3.01 for the variance analysis and the correlation analysis by SAS 9.0 according to different requirements was done. Quantitative analyses were presented as mean values and the reproducibility of the results was expressed as standard error (S.E.).

## 4. Conclusions

In summary, Ac-SPS and Ac-SuSy activities are differentially responsive to sugar dynamic characteristics and show contrasting expression patterns consistent with different functional roles. The phenomena are expected to shed further light on the important horticultural phenomenon of sugar accumulation that determines fruit quality. A cycle of sucrose breakdown in the cytosol of sink tissues could be mediated to keep the balance of sugars through both Ac-SuSy and Ac-NI. In addition, we also conclude that Ac-NI could be involved in regulating crucial steps of plant development or at least play a supportive role to other sugar metabolism enzymes by providing substrates to the cells and by generating sugar signals in a temporally and spatially restricted fashion.

## Figures and Tables

**Table 1:** Primer sets used for PCR amplification and expression analysis.

Primer name	Forward and reverse primes (5′-3′)	Tm (°C)
*Ac-sps* degenerate primers	GGA(G/A)CTTGG(T/A/G/C)CG(T/G)GATTCTGATA CAGGAAC(A/T/C)TC(A/T)GA(C/T)TG(C/T)TTGTG	52
*Ac-sps* specific primers	GCTACGGCGAGCCAACCGAGAT GTGGCCAAGAGCGCCGTCAAC	60
*Ac-susy* specific primers 1	GGATGACCGGTCTAAGCCCATCAT CCATTTCACGCAGTTTGGCGCAT	60
*Ac-ni* degenerate primers	CAG(T/C)T(T/G/A)CAGAG(C/T)TGGGAGA GTGTC(A/G)TA(A/C)TA(T/C)TCAGGCCA	55
*Ac-ni* specific primers	GCTCTGGGGATTTGTCAGTTCAG CGATCAATCATGCACGAGCCGT	60
18S rRNA specific primers 2	GCCGGCGACGCATCATTCAA CGCGCCTGCTGCCTTCCTTG	60

1 Oligonucleotides designed based on the *Ac-susy* sequence from pineapple fruit (GenBank ID: DQ438976);

2 Oligonucleotides designed based on the 18S rRNA sequence from pineapple (GenBank ID: D29786).
